# Significant race and gender differences in anterior cruciate ligament tibial footprint location: a 3D-based analysis

**DOI:** 10.1186/s10195-023-00710-w

**Published:** 2023-06-30

**Authors:** Lihang Zhang, Changzhao Li, Jiaying Zhang, Diyang Zou, Dimitris Dimitriou, Xing Xing, Tsung-Yuan Tsai, Pingyue Li

**Affiliations:** 1 Guangdong Key Lab of Orthopedic Technology and Implant, General Hospital of Southern Theater Command of PLA,The First School of Clinical Medicine, Southern Medical University, Guangzhou, China; 2 Department of Graduate School, Guangzhou University of Chinese Medicine, Guangzhou, China; 3 School of Biomedical Engineering and Med-X Research Institute, Shanghai Jiao Tong University, Shanghai, China; 4 Engineering Research Center of Digital Medicine and Clinical Translation, Ministry of Education, Shanghai, China; 5 Shanghai Key Laboratory of Orthopedic Implants and Clinical Translational R&D Center of 3D Printing Technology, Department of Orthopedic Surgery, Shanghai Ninth People’s Hospital, Shanghai Jiao Tong University School of Medicine, Shanghai, China; 6 Department of Orthopedics, University Hospital Balgrist, Zurich, Switzerland; 7 Department of Biostatistics, Bioinformatics and Biomathematics, Georgetown University, N.W. Washington, DC USA; 8 Department of Social Medicine and Health Management, School of Public Health, Peking University, Beijing, People’s Republic of China

**Keywords:** Anterior cruciate ligament, Ethnicity, Gender, Tibial attachment, Single-bundle reconstruction, Landmarks, Iatrogenic injury

## Abstract

**Background:**

The aim of the present study was to identify potential race- or gender-specific differences in anterior cruciate ligament (ACL) tibial footprint location from the tibia anatomical coordinate system (tACS) origin, investigate the distances from the tibial footprint to the anterior root of the lateral meniscus (ARLM) and the medial tibial spine (MTS), determine how reliable the ARLM and MTS can be in locating the ACL tibial footprint, and assess the risk of iatrogenic ARLM injuries caused by using reamers with various diameters (7–10 mm).

**Patients and methods:**

Magnetic resonance images of 91 Chinese and 91 Caucasian subjects were used for the reconstruction of three-dimensional (3D) tibial and ACL tibial footprint models. The anatomical coordinate system was applied to reflect the anatomical locations of scanned samples.

**Results:**

The average anteroposterior (A/P) tibial footprint location was 17.1 ± 2.3 mm and 20.0 ± 3.4 mm in Chinese and Caucasians, respectively (*P* < .001). The average mediolateral (M/L) tibial footprint location was 34.2 ± 2.4 mm and 37.4 ± 3.6 mm in Chinese and Caucasians, respectively (*P* < .001). The average difference between men and women was 2 mm in Chinese and 3.1 mm in Caucasians. The safe zone for tibial tunnel reaming to avoid ARLM injury was 2.2 mm and 1.9 mm away from the central tibial footprint in the Chinese and Caucasians, respectively. The probability of damaging the ARLM by using reamers with various diameters ranged from 0% for Chinese males with a 7 mm reamer to 30% in Caucasian females with a 10 mm reamer.

**Conclusions:**

The significant race- and gender-specific differences in the ACL tibial footprint should be taken in consideration during anatomic ACL reconstruction. The ARLM and MTS are reliable intraoperative landmarks for identifying the tibial ACL footprint. Caucasians and females might be more prone to iatrogenic ARLM injury.

Level of evidence: III, cohort study.

*Trial registration:* This study has been approved by the ethical research committee of the General Hospital of Southern Theater Command of PLA under the code: [2019] No.10.

## Introduction

It is estimated that more than 2 million ACL ruptures occur worldwide every year [1, 2]. The main concept of anatomic ACL reconstruction is to restore the native ACL attachments and function, thereby achieving better clinical outcomes than nonanatomic ACL reconstruction [3, 4]. Accurate tunnel placement requires an excellent knowledge of the ACL attachment anatomy. Despite extensive literature describing the location of the femoral footprint [5–7], tibial-insertion-related data are limited and mostly based on small-sized cadaveric studies focusing on elderly unpaired subjects from a single ethnic background and using two-dimensional techniques [8–11].

More importantly, anatomical studies on ACL tibial attachment in the Chinese population seem to be inadequate, even though a large number of ACL injuries occur annually in China. Moreover, the placement of the tibial tunnel for anatomic single-bundle ACL reconstruction was developed based on the anatomical features of the Caucasian population [5, 12–15]. So, understanding the differences in ACL tibial footprint between Chinese and Caucasian populations may help surgeons to develop ethnically specific surgical strategies to achieve satisfactory outcomes. The spatial distribution of the ARLM and MTS should be investigated based on a large sample of the Chinese population so that the tibial tunnel in Chinese patients can be identified and iatrogenic ARLM injury prevented. Furthermore, reliable landmarks are necessary to guide the surgeon during tibial tunnel reaming, especially in the setting of revision surgeries, where the ACL stump is not available.

Since several race- and gender-specific anatomical differences in healthy knees have been described [16], it was assumed that the ACL tibial footprint in Chinese differs significantly from that in Caucasian individuals. Thus, the aims of the present study were (1) to investigate the three-dimensional morphological differences in ACL tibial attachment between Chinese and Caucasian individuals with intact ACLs, (2) to determine the spatial distributions of the ARLM and MTS in different racial populations; (3) to examine the reliability of ARLM and MTS for determining tibial tunnel placement, and (4) to calculate the percentages of iatrogenic injuries to the ARLM caused by reamers with various diameters, which allows the safe zone to avoid damage to the ARLM to be defined.

## Materials and methods

### Patient selection and study design

After the approval of the ethics committees, we obtained the informed consent of 91 Chinese and 91 Caucasian patients with intact ACLs to review their medical records and the magnetic resonance imaging (MRI) database. The subjects were healthy people who participated in a physical examination between 2019 and 2022. Since 91 Caucasian subjects (61 men, 30 women) were seen for physical examinations during this period, we used a random sampling method to select 91 Chinese subjects from the same period. The average ages of the Chinese and Caucasian groups were 25.3 and 25.8 years old (*p* = 0.2533), and the average body mass index (BMI) values were 22.5 kg/m^2^ and 22.7 kg/m^2^ (*p* = 0.6976), respectively. The inclusion criteria were an age of less than 45 years and intact ACLs. Exclusion criteria included lateral meniscus anterior root tears, previous surgery, symptoms in the affected knee, or a poor-quality MRI image that could not be used to identify and reconstruct the ACL footprint.

### Magnetic resonance parameters and protocols

**Fig. 1:**
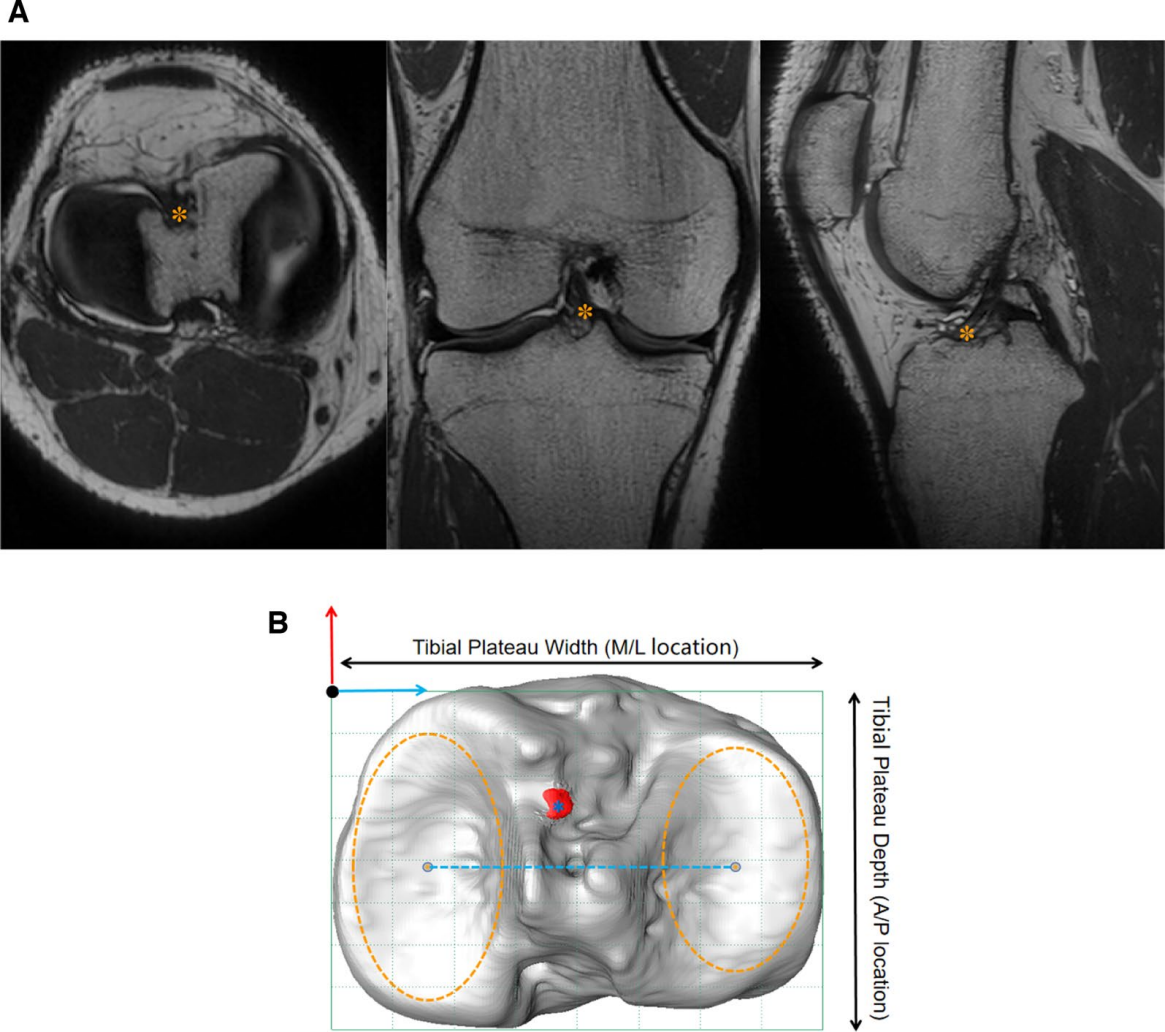
**a** 3D MRI clearly showing the ACL in three orientations. The ACL tibial footprint location is marked with an* orange star*.** b** Three-dimensional surface models of the right tibia and the tibial footprint area were reconstructed. The anatomical coordinate system of the tibia (tACS) was also created. The origin is shown with a* black dot*, and the medial/lateral axis connecting the centers of the best-fitted ellipses on the articular surfaces of the medial and lateral tibial plateaus is shown with a* blue line*. The anterior/posterior axis is shown with a* red arrow*. Within the best-fitted plane, a bounding box was defined by the depth and width of the tibial plateau. The origin of the tACS was then moved to the most anterior and medial point of the bounding box

Three-dimensional (3D) MRI, a method proven to deliver clearer, more accurate results [17], was used to obtain a vivid reflection of the ACL. All enrolled subjects were scanned by a 3.0-T MRI system with fully extended knees (Skyra; Siemens). Proton density 3D fast spin-echo volume sequences (PD space) were applied to collect images (slice thickness: 0.50 mm, voxel size: 0.3 × 0.3 × 0.5 mm) (Fig. 1a). Amira 6.5 FEI SVG (Thermo Fisher, USA) was used to reconstruct a 3D model of the tibia containing the meniscus according to a proven and publicly available method [18] (Fig. 1b). Reconstruction of the ACL tibial attachment area was performed using a validated and published method [19, 20]. The measurement of parameters related to the tibial models was completed by MATLAB 2014.

### Concept of the proximal tibial coordinate system

The tibial anatomical coordinate system (tACS) was developed based on a previously published method [21, 22]. The medial and lateral tibial plateaus were best fitted and two fitted ellipses were generated. The mediolateral (M/L) axis was parallel to the line connecting the two fitted ellipses. The anteroposterior (A/P) axis was perpendicular to both the M/L axis and the proximal tibial long axis. The width and depth of the tibial plateau were defined as the maximum distances in the M/L and A/P directions, respectively. The origin of the coordinate system was located at the most anteromedial point of the grid (Fig. 1b).

### Description of measurement indicators

The coordinates of the central tibial footprint were represented by the distances of the footprint from the origin in the M/L and A/P directions. As shown in Stäuebli and Rauschning’s method [10], the normalized location of the tibial footprint corresponded to the distances in the M/L and A/P directions separated by the width and depth of the tibial plateau, respectively. The distances from the tibial footprint to the ARLM and MTS were also measured in the M/L and A/P directions. Positive values represented more anterior and lateral positions.

### Adjustment of normalized locations for consistency analysis

**Fig. 2:**
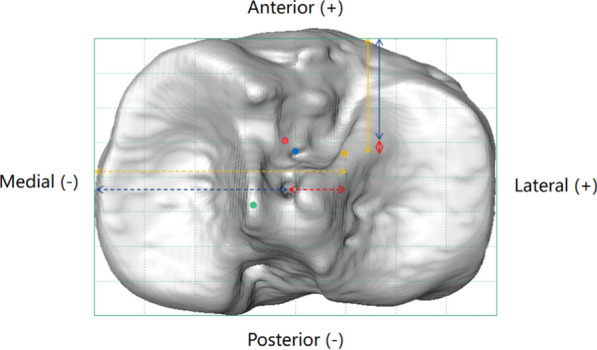
Axial view of a right knee demonstrating the normalized tibial ACL footprint location in Chinese (*red dot*) and Caucasian (*blue dot*) patients in the A/P and M/L directions. The most posteromedial point of the ARLM is indicated by a* yellow dot*. The adjusted normalized A/P (*blue arrow*) and M/L (*dashed blue arrow*) tibial footprint locations according to the ARLM were defined as the average normalized A/P (*yellow arrow*) and M/L (*dashed yellow arrow*) distances of the ARLM from the origin of the tACS minus the normalized A/P (*red arrow*) and M/L (*dashed red arrow*) distances of the tibial footprint from the ARLM, respectively. *ACL* anterior cruciate ligament, *ARLM* anterior root of the lateral meniscus, *A/P* anteroposterior, *M/L* mediolateral, *tACS* tibial anatomical coordinate system

**Fig. 3:**
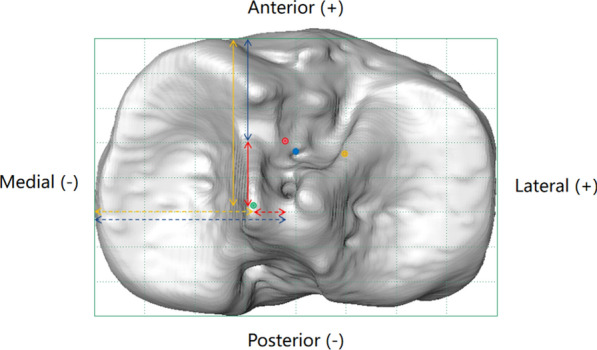
Axial view of a right knee demonstrating the normalized tibial ACL footprint location in Chinese (*red dot*) and Caucasian (*blue dot*) patients in the A/P and M/L directions. The peak of the MTS is shown with a* green dot*. The adjusted normalized A/P tibial footprint location according to the MTS (*blue arrow*) was defined as the average normalized A/P distance of the MTS from the origin of the tACS (*yellow arrow*) minus the normalized A/P distance of the tibial footprint from the MTS (*red arrow*). The adjusted normalized M/L tibial footprint location according to the MTS (*dashed blue arrow*) was defined as the average normalized M/L distance of the MTS from the origin of the tACS (*dashed yellow arrow*) plus the normalized A/P distance of the tibial footprint from the MTS (*dashed red arrow*).* ACL* anterior cruciate ligament,* A/P* anteroposterior,* MTS* medial tibial spine,* M/L* mediolateral,* tACS* tibial anatomical coordinate system

To find out whether the distances from the different reference points (ARLM, MTS, and tACS) to the ACL footprint center were statistically highly consistent, the parameters were adjusted as follows. Regarding the ARLM, the adjusted location of the normalized tibial footprint equaled the distance from the normalized ARLM location to the origin minus the distance from the normalized ARLM location to the tibial footprint in the A/P and M/L directions, respectively (Fig. 2). Similarly, for the MTS, the adjusted location of the normalized A/P tibial footprint equaled the distance from the normalized A/P MTS location to the origin minus the distance from the normalized A/P MTS location to the tibial footprint (Fig. 3), and the adjusted location of the normalized M/L tibial footprint equaled the distance from the normalized M/L MTS location to the origin plus the distance from the normalized M/L MTS location to the tibial footprint (Fig. 3).

### Risk assessment of ARLM injury

**Fig. 4:**
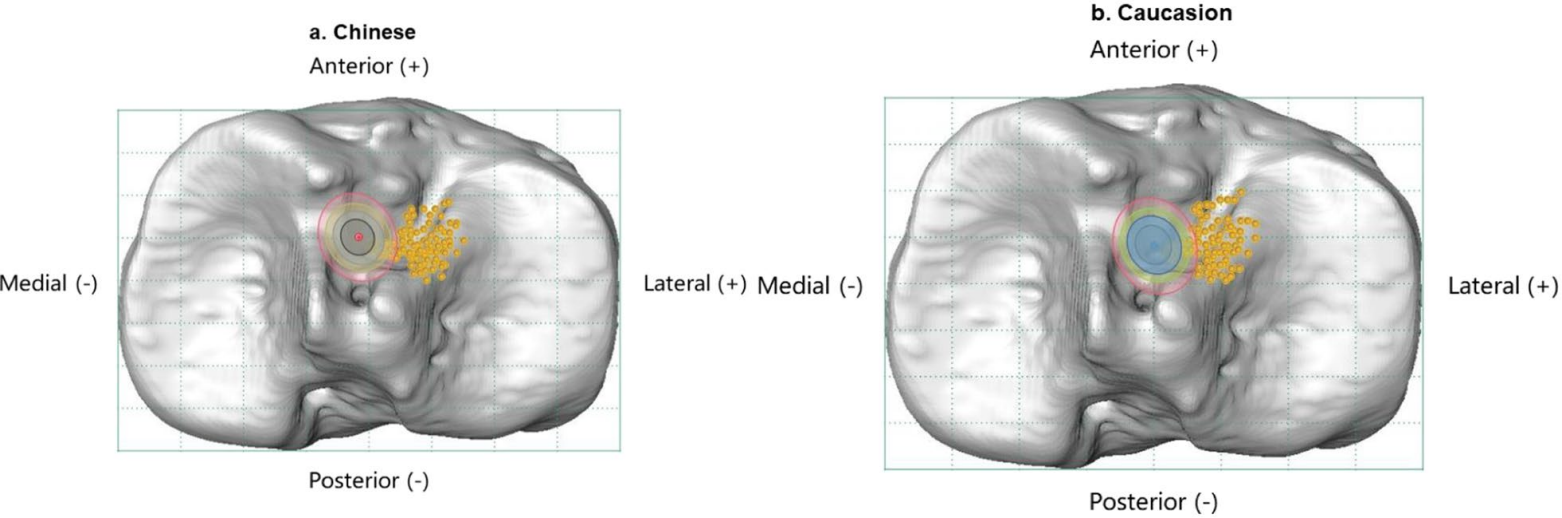
Two diagrams showing the relationship between the ARLM and reamers with different diameters. The* orange dots* indicate the positions of the ARLMs. The* red and blue dots* represent the centers of the ACL tibial footprints. The* gray circle*,* blue circle*,* green circle*, and* red circle* represent tunnel areas with diameters of 7 mm, 8 mm, 9 mm, and 10 mm, respectively

**Fig. 5:**
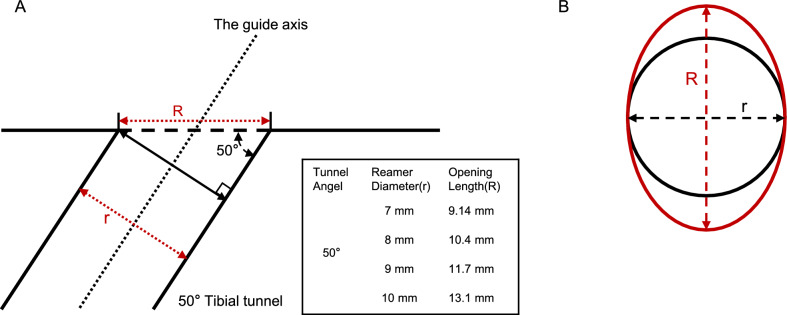
**A** Sagittal view of a 50° tibial tunnel, and the opening lengths formed by reamers with different diameters (*R* is the long axis of the elliptical tunnel opening). **B** Axial view of the tibial tunnel opening (*red ellipse*). *R* = * r*/sin(50°)

With the tibial footprint center of each subject taken as the center, concentric ellipses with short axes (*r*) of 3.5 mm, 4 mm, 4.5 mm, and 5 mm were drawn to simulate reamers with various diameters. The long axis of the ellipse (*R*) was dependent on the drilling angle of the tibial tunnel, and was a certain formula relating *R* and *r*: *R* = *r*/sin(tunnel angle) [53]. Based on literature reports and our surgical experience, we set the tibial tunnel angle to 50° [53, 54]. Since surgeons also choose different angles between the drill and the tibial sagittal plane, the direction of the long axis of the elliptic opening on the tibial plateau is variable. Therefore, if the distance between the footprint center and ARLM was less than the axis length of the ellipse (including the long and short axes), it was judged as indicating an ARLM injury. Furthermore, the risk of damage to the ARLM was between the probabilities caused by the two different lengths (*r* and* R*) (Figs. 4a, b, 5a, b).

### Reliability analysis

The ARLM, ACL footprint, and the MTS were sketched manually on the original images. The intra- and interobserver reliabilities of these parameters were assessed based on single-measure intraclass correlation coefficients (ICCs) by two physicians (Zhang and Lee) who did not know anything about the study subjects.

### Statistical analysis

G*Power version 3.1 (Franz Faul, Uni Kiel, Germany) was used in the post-hoc power analysis for statistical power (1 −* β*) estimation, with a medium effect size and *a* = 0.05. The Kolmogorov–Smirnov test was conducted to inspect the sample normality. If the sample was well fitted by a normal distribution, we used the two-sided* t*-test. Otherwise, Wilcoxon's signed-rank test was required for non-parametric statistics. Bland–Altman plots were utilized to analyze the consistency between the tibial attachment positions with reference to the ARLM, MTS, and the origin of the coordinate system [23]. Additionally, the correlation between the tibial attachment positions determined by the ARLM, MTS, and the origin of the tACS was analyzed with the Pearson correlation coefficient. SPSS software (version 23, IBM Inc., Chicago, IL, USA) was used to perform all data analyses. A result with *p* < 0.05 was considered statistically significant. The safety zone for preventing damage to the ARLM caused by reamers with different radii during the tibial tunnel expansion was the average distance from the ARLM to the tibial attachment center minus double the standard deviation.

## Results

### Power analysis and reliability analysis

The statistical power needed to identify differences in the locations of the tibial footprint between Chinese (*n* = 91) and Caucasians (*n* = 91) was 91.6%. The intra- and interobserver ICCs of all the groups fell within the range 0.80–0.95.

### Tibial footprint position with respect to the origin of the tACS

The average depth and width of the tibial plateau in Chinese were 54.3 ± 4.2 mm and 75.7 ± 5.8 mm, respectively; for Caucasians, the results were 56.7 ± 5.2 mm and 78.1 ± 6.6 mm. In terms of racial differences, the location of the tibial footprint in the Chinese population was 17.1 ± 2.3 mm and 34.2 ± 2.4 mm of the tibial plateau depth and width on average, respectively (Table 1). In the Caucasian population, the tibial attachment was located at 20.0 ± 3.4 mm and 37.4 ± 3.6 mm of the tibial plateau width and depth on average, respectively (Table 1). In terms of gender differences, the tibial footprint was located at a depth of 17.7 ± 2.6 mm and a width of 35.7 ± 1.8 mm in Chinese males, and at a depth of 15.7 ± 1.8 mm and a width of 31.7 ± 1.7 mm in Chinese females (Table 2). For the Caucasian population, the tibial footprint was located at a depth of 21.0 ± 3.2 mm and a width of 39.0 ± 2.9 mm in Caucasian males, and a depth of 17.9 ± 2.4 mm and a width of 34.2 ± 2.6 mm in Caucasian females (Table 3).

**Table 1:** The average anatomical location of the tibial ACL footprint in Chinese and Caucasians

Reference point	Measurement direction and units	ACL footprint location (mean ± sd)		*P* value
		Chinese	Caucasian	
tACS	A/P (mm)	17.1 ± 2.3	20.0 ± 3.4	< 0.001***
	M/L (mm)	34.2 ± 2.4	37.4 ± 3.6	< 0.001***
	Normalized A/P (%)	31.5 ± 4.2	35.3 ± 6.0	< 0.001***
	Normalized M/L (%)	45.2 ± 3.2	47.9 ± 4.6	< 0.001***
Posteromedial ARLM	A/P (mm)	1.4 ± 2.1	1.2 ± 2.3	= 0.543
	M/L (mm)	8.2 ± 3.0	6.7 ± 2.4	< 0.001***
	Normalized A/P (%)	2.6 ± 3.9	2.1 ± 4.1	= 0.403
	Normalized M/L (%)	10.8 ± 4.0	8.6 ± 3.1	< 0.001***
Peak of MTS	A/P (mm)	13.2 ± 2.8	11.6 ± 2.1	< 0.001***
	M/L (mm)	4.3 ± 1.5	5.2 ± 2.4	= 0.003**
	Normalized A/P (%)	24.3 ± 5.2	20.5 ± 3.7	< 0.001***
	Normalized M/L (%)	5.7 ± 2.0	6.7 ± 3.1	= 0.011*

*ACL* anterior cruciate ligament, *A/P* anteroposterior, *M/L* mediolateral,* ARLM* Anterior root of the lateral meniscus, *MTS* medial tibial spine, *tACS* tibial anatomical coordinate system

* 0.01 < *P* < 0.05; **, 0.001 < *P*< 0.01; ***, *P* < 0.001

**Table 2:** The average anatomical location of the tibial ACL footprint in Chinese males and females

Reference point	Measurement direction and units	ACL footprint location (mean ± sd)		*P* value
		Chinese males	Chinese females	
tACS	A/P (mm)	17.7 ± 2.6	15.7 ± 1.8	< 0.001***
	M/L (mm)	35.7 ± 1.8	31.7 ± 1.7	< 0.001***
	Normalized A/P (%)	32.1 ± 4.7	30.0 ± 3.4	= 0.034*
	Normalized M/L (%)	45.7 ± 2.3	44.6 ± 2.4	= 0.042**
Posteromedial ARLM	A/P (mm)	0.9 ± 2.0	2.1 ± 2.1	= 0.012*
	M/L (mm)	9.4 ± 2.9	6.4 ± 2.2	< 0.001***
	Normalized A/P (%)	1.6 ± 3.6	4.0 ± 4.0	= 0.008**
	Normalized M/L (%)	15.7 ± 3.3	9.0 ± 3.1	< 0.001***
Peak of MTS	A/P (mm)	13.6 ± 2.9	12.7 ± 2.6	= 0.141
	M/L (mm)	4.7 ± 1.3	4.6 ± 1.4	= 0.749
	Normalized A/P (%)	24.6 ± 5.3	24.2 ± 5.0	= 0.727
	Normalized M/L (%)	6.0 ± 1.7	6.5 ± 2.0	= 0.246

*ACL* anterior cruciate ligament, *A/P* anteroposterior, *M/L* mediolateral, *ARLM* anterior root of the lateral meniscus, *MTS* medial tibial spine, *tACS* tibial anatomical coordinate system

* 0.01 < *P* < 0.05; ** 0.001 < *P* < 0.01; *** *P* < 0.001

**Table 3:** The average anatomical location of the tibial ACL footprint in Caucasian males and females

Reference point	Measurement direction and units	ACL footprint location (mean ± sd)		*P* value
		Caucasian males	Caucasian females	
tACS	A/P (mm)	21.0 ± 3.2	17.9 ± 2.4	< 0.001***
	M/L (mm)	39.0 ± 2.9	34.2 ± 2.6	< 0.001***
	Normalized A/P (%)	35.8 ± 5.5	34.0 ± 4.6	= 0.106
	Normalized M/L (%)	48.0 ± 3.6	47.7 ± 3.6	= 0.711
Posteromedial ARLM	A/P (mm)	1.1 ± 2.4	1.2 ± 2.2	= 0.844
	M/L (mm)	7.4 ± 2.3	5.3 ± 2.2	< 0.001***
	Normalized A/P (%)	1.9 ± 4.1	2.2 ± 4.2	= 0.749
	Normalized M/L (%)	9.1 ± 2.8	7.4 ± 3.1	= 0.014*
Peak of MTS	A/P (mm)	11.6 ± 2.0	11.7 ± 2.4	= 0.844
	M/L (mm)	5.2 ± 2.4	5.2 ± 2.4	= 0.970
	Normalized A/P (%)	19.8 ± 3.4	22.2 ± 4.6	= 0.015*
	Normalized M/L (%)	6.4 ± 3.0	7.3 ± 3.3	= 0.214

*ACL* anterior cruciate ligament, *A/P* anteroposterior, *M/L* mediolateral, *ARLM* anterior root of the lateral meniscus, *MTS* medial tibial spine, *tACS* tibial anatomical coordinate system

* 0.01 < *P* < 0.05; ** 0.001 < *P* < 0.01; *** *P* < 0.001

### Tibial footprint position with respect to the ARLM

The A/P tibial footprint in Chinese and Caucasians was located at an average of 1.4 ± 2.1 mm and 1.2 ± 2.3 mm anterior to the ARLM, respectively (*p* = 0.403). The M/L tibial footprint in the studied population was situated at an average of 8.2 ± 3.0 mm and 6.7 ± 2.4 mm medial to the ARLM, respectively (*p* < 0.001) (Table 1). To avoid damaging the ARLM, the safety zone for Chinese patients was 2.2 mm lateral to the footprint center and 1.9 mm lateral to the footprint center in Caucasians.

### Tibial footprint position with respect to the MTS

The A/P tibial footprint in Chinese and Caucasians was situated at an average of 13.2 ± 2.8 mm and 11.6 ± 2.1 mm anterior to the MTS, respectively (*p* < 0.001). The M/L tibial footprint in Chinese and Caucasians was located at an average of 4.3 ± 1.5 mm and 5.2 ± 2.4 mm medial to the MTS, respectively (*p* = 0.011) (Table 1).

### Consistency between the tibial footprint positions referencing the ARLM, MTS, and tACS

According to the result of the Bland–Altman plot, the consistency of the M/L and A/P tibial attachment positions obtained when the ARLM and tACS were used as reference points was quantified as an average bias of − 1.7% (95% limits of agreement: − 9.8 to 6.2%) and 0.001% (95% limits of agreement: − 5.2 to 5.2%), respectively. Similarly, the consistency of the M/L and A/P tibial footprint positions obtained when the MTS and tACS were used as reference points was quantified as an average bias of 0.006% (95% limits of agreement: − 3.9 to 3.9%) and 0.003% (95% limits of agreement: − 7.4 to 7.4%), respectively. The Pearson’s correlation coefficients between the tibial footprint positions referencing the ARLM and tACS were *r* = 0.61 (*p* < 0.001) and *r* = 0.80 (*p* < 0.001), while those between the tibial footprint locations referencing the MTS and tACS were *r* = 0.51 (*p* < 0.001) and* r * = 0.86 (*p* < 0.001).

### Risk of iatrogenic ARLM injury with different reamers

The results showed that Caucasians and females were more prone to ARLM injury during the reaming of the tibial tunnel. The risk of ARLM injury when using reamers with various diameters ranged from 0% for Chinese males with a 7 mm reamer to 70% in Caucasian females with a 10 mm reamer. The risk was almost 2–5 times higher for Caucasians than for Chinese, and 2–3 times higher for females than for males (Table 4).

**Table 4:** Risk of injuring the ARLM using reamers with different diameters and the safety zone

		Diameter								Safety zone (mm)
		7 mm		8 mm		9 mm		10 mm		
		*r*	*R*	*r*	*R*	*r*	*R*	*r*	*R*	
Patients	Chinese	2/91 (2.2%)	7/91 (7.7%)	5/91 (5.5%)	11/91 (12.1%)	7/91 (7.7%)	14/91 (15.4%)	10/91 (11.0%)	19/91 (20.9%)	2.2
	Male	0/61 (0.00%)	3/61 (4.9%)	2/61 (3.3%)	4/61 (6.6%)	3/61 (4.9%)	6/61 (9,8%)	4/61 (6.6%)	9/61 (14.8%)	
	Female	2/30 (6.7%)	4/30 (13.3%)	3/30 (10.0%)	7/30 (23.3%)	4/30 (13.3%)	8/30 (26.7%)	6/30 (20.0%)	10/30 (33.3%)	
	Caucasian	9/91 (9.9%)	18/91 (19.8%)	14/91 (15.4%)	24/91 (26.4%)	18/91 (19.8%)	31/91 (34.1%)	21/91 (23.1%)	39/91 (42.9%)	1.9
	Male	4/61 (6.6%)	9/61 (14.8%)	6/61 (9.8%)	12/61 (19.7%)	9/61 (14.8%)	13/61 (21.3%)	12/61 (19.7%)	18/61 (29.5%)	
	Female	5/30 (16.7%)	9/30 (30.0%)	8/30 (26.7%)	12/30 (40.00%)	9/30 (30.0%)	18/30 (60.00%)	9/30 (30.00%)	21/30 (70.00%)	

*ARLM* anterior root of the lateral meniscus, *r* diameter of the reamer (7, 8, 9, or 10 mm), *R* = *r*/sin(50°)

## Discussion

The most important finding of the present study was that significant race- and gender-specific differences exist regarding the location of the tibial ACL footprint. Furthermore, the MTS and ARLM may be reliable arthroscopic landmarks for identifying the tibial ACL footprint. The safe zone for tibial tunnel reaming to avoid ARLM injury was 2.2 mm and 1.9 mm from the central tibial footprint in Chinese and Caucasians, respectively. The probability of damaging the ARLM by using reamers with various diameters ranged from 0% for Chinese males with a 7 mm reamer to 70% in Caucasian females with a 10 mm reamer when the tunnel is drilled at a 50° angle.

For ACL single-bundle reconstruction surgery, the current surgical strategy is to pinpoint the anatomical center of the tibial footprint [24–26, 50, 51]. Edwards et al. [27] and Staübli et al. [10] reported cadaveric studies in which the tibial attachment was situated at 17 ± 5 mm (36.0% ± 5.0%) and 21 ± 2.6 mm (41.2%) in the A/P direction and at 37 ± 4 mm (46.3% ± 5.0%) in the M/L direction on average, respectively. The results obtained in the present study were similar to theirs (Tables 1, 2, and 3), but the normalized location was more anterior and medial in the Chinese group, especially in females. According to previous research findings [9, 28, 29], the anterior medial bundle is vital for maintaining the anterior stability of the knee joint. A more anterior and medial tunnel tremendously increases the risk of graft rupture. Our results may explain why Chinese and female athletes were more vulnerable to ACL injuries [30–35, 52]. Meanwhile, the statistical differences in Tables 1 and 2 suggest that ethnicity and gender may be important factors in locating the tibial tunnel, which might have been underestimated by surgeons for a long time.

Finding suitable intraoperative landmarks to use to locate the tibial tunnel holds the key to this surgery, especially when the ACL stump is not available (for example, after revision and conservative treatment failure). Edwards et al. [27] considered the MTS to be a reliable landmark only in the M/L direction, and the average distance from the tibial attachment to the MTS was 5.0 ± 1.0 mm. Kassam et al. [36] reported that, in their study of two-dimensional MRI images, the tibial attachment was situated 0.1 mm posterior to the ARLM on average. Having observed 20 unpaired knee specimens, Zantop et al. [37] summarized that the AM bundle center was in alignment with the ARLM while the PL bundle center was 11.2 ± 1.2 mm posterior and 4.1 ± 0.6 mm medial to the ARLM. After studying 8 knee joints from cadaveric specimens with an average age of 65.8 years, Ferretti et al. [38] pointed out that the MTS was a reliable landmark only in the A/P direction, and the location of the tibial attachment center was 5.7 ± 1.1 mm anterior to the MTS on average. However, they believed that the relationship between the ARLM and the ACL footprint was variable. Hutchinson et al. [39] reported that, in their study of 42 coupled cadaver knees, the distance between the posterior edge of the tibial attachment and the MTS was 8.8 ± 2.0 mm on average. In the present study, we confirmed that both the ARLM and the MTS are reliable landmarks for locating the ACL tibial tunnel. In particular, the ARLM is an easily identifiable anatomical landmark under the arthroscope, suggesting that it could be used as a more available intraoperative indicator in anatomical single-bundle ACL reconstruction.

According to previous studies [40, 41], the minor axis of the native tibial footprint (5.2 ± 0.4 mm) was far shorter than the tunnel diameter. The ARLM attachment forming the lateral edge of the tibial footprint is vulnerable to damage [42, 43]. Iatrogenic injury may disintegrate the ARLM attachment, compromise the strength of the ARLM attachment site, and eventually accelerate the degeneration of the knee joints [44, 45]. Watson et al. [46] announced that the risk of ARLM injury caused by a 10-mm reamer was 66%. Oishi et al. [47] noted that reaming the tunnel with a 10-mm reamer resulted in damage to 21.7% of the ARLMs in specimens. LaPrade et al. [40] reported that, when using an 11-mm reamer, the ARLMs in all specimens were injured. However, a tunnel of such a size is rarely seen in our ACL surgeries. We found that the probability of damaging the ARLM when using reamers with different diameters ranged from 0 to 70.00%, which was similar to previous reports (Table 4). However, results showed that injuries to the ARLM may be more likely to occur in Caucasians and females, which was not recognized in previous studies (Fig. 4). Also, the safety zone used to prevent damage to the ARLM during tibial tunnel reaming was 2.2 mm and 1.9 mm lateral to the tibial footprint center in Chinese and Caucasian populations, respectively. We believe that the size of the tibial plateau is slightly larger in Caucasians, but the center of the ACL footprint is further away from both the ARLM attachment and the MTS in Chinese. This indicates that these two anatomical landmarks may be more dispersed on the tibial plateau in the Chinese population, which explains why the Chinese population had a larger safety zone and a lower risk of ARLM injury.

The present study should be interpreted in light of its potential limitations. Firstly, MRI-based 3D models were used to identify the anatomy of the tibial ACL footprint and the ARLM. Even though cadaveric studies are considered the gold standard, 3D MRI has proven to be an accurate and reliable imaging method [17, 48, 49] and could use a larger sample size. Moreover, since the subjects in this study were Chinese and Caucasian, the results do not apply to African Americans, meaning that further studies with greater racial diversity are needed. Furthermore, the long axis of the elliptical tunnel opening (*R*) was also dependent on the drilling angle, so we used a certain range to report the injury risk.

## Conclusion

There are significant race- and gender-specific differences in the tibial ACL footprint. The ARLM and MTS may be reliable arthroscopic landmarks for identifying the tibial ACL footprint during anatomical ACL reconstruction. The safe zone needed to avoid damage to the ARLM during tibial tunnel reaming was 2.2 mm and 1.9 mm lateral to the central tibial footprint in Chinese and Caucasians, respectively. The probability of damaging the ARLM by using reamers with various diameters ranged from 0% for Chinese males with a 7 mm reamer to 70% in Caucasian females with a 10 mm reamer when the tunnel was drilled at a 50° angle.

## Data Availability

The datasets used and/or analyzed during the current study are available from the corresponding author on reasonable request.

## Abbreviations

ACL: Anterior cruciate ligament
tACS: Tibia anatomical coordinate system
ARLM: Anterior root of the lateral meniscus
MTS: Medial tibial spine
3D: Three-dimensional
MRI: Magnetic resonance imaging
M/L: Mediolateral
A/P: Anteroposterior
SPSS: Statistical Package for the Social Sciences
ICCs: Intraclass correlation coefficients

## References

[1] Spindler KP, Wright RW (2008). Anterior cruciate ligament tear. New Engl J Med.

[2] Renström PA (2013). Eight clinical conundrums relating to anterior cruciate ligament (ACL) injury in sport: recent evidence and a personal reflection: Table 1. Brit J Sport Med.

[3] Hussein M, van Eck CF, Cretnik A, Dinevski D, Fu FH (2012). Prospective randomized clinical evaluation of conventional single-bundle, anatomic single-bundle, and anatomic double-bundle anterior cruciate ligament reconstruction: 281 cases with 3- to 5-year follow-up. Am J Sports Med.

[4] Marchant BG, Noyes FR, Barber-Westin SD, Fleckenstein C (2010). Prevalence of nonanatomical graft placement in a series of failed anterior cruciate ligament reconstructions. Am J Sports Med.

[5] Colombet P, Robinson J, Christel P (2006). Morphology of anterior cruciate ligament attachments for anatomic reconstruction: a cadaveric dissection and radiographic study. Arthroscopy.

[6] Luites J, Verdonschot N (2017). Radiographic positions of femoral ACL, AM and PL centres: accuracy of guidelines based on the lateral quadrant method. Knee Surg Sports Traumatol Arthrosc.

[7] Yamamoto Y, Hsu W, Woo SL (2004). Knee stability and graft function after anterior cruciate ligament reconstruction: a comparison of a lateral and an anatomical femoral tunnel placement. Am J Sports Med.

[8] Heming JF, Rand J, Steiner ME (2007). Anatomical limitations of transtibial drilling in anterior cruciate ligament reconstruction. Am J Sports Med.

[9] Petersen W, Zantop T (2007). Anatomy of the anterior cruciate ligament with regard to its two bundles. Clin Orthop Relat Res.

[10] Staubli HU, Rauschning W (1994). Tibial attachment area of the anterior cruciate ligament in the extended knee position. Anatomy and cryosections in vitro complemented by magnetic resonance arthrography in vivo. Knee Surg Sports Traumatol Arthrosc..

[11] Smith JO, Yasen S, Risebury MJ, Wilson AJ (2014). Femoral and tibial tunnel positioning on graft isometry in anterior cruciate ligament reconstruction: a cadaveric study. J Orthop Surg (Hong Kong).

[12] Luites JW, Wymenga AB, Blankevoort L, Kooloos JG (2007). Description of the attachment geometry of the anteromedial and posterolateral bundles of the ACL from arthroscopic perspective for anatomical tunnel placement. Knee Surg Sports Traumatol Arthrosc.

[13] Purnell ML, Larson AI, Clancy W (2008). Anterior cruciate ligament insertions on the tibia and femur and their relationships to critical bony landmarks using high-resolution volume-rendering computed tomography. Am J Sports Med.

[14] Jackson DW, Gasser SI (1994). Tibial tunnel placement in ACL reconstruction. Arthroscopy.

[15] Marwan Y, Bottcher J, Laverdiere C (2020). Three-dimensional magnetic resonance imaging for guiding tibial and femoral tunnel position in anterior cruciate ligament reconstruction: a cadaveric study. Orthop J Sports Med.

[16] Dimitriou D, Wang Z, Zou D, Tsai TY, Helmy N (2019). The femoral footprint position of the anterior cruciate ligament might be a predisposing factor to a noncontact anterior cruciate ligament rupture. Am J Sports Med.

[17] Araki D, Thorhauer E, Tashman S (2018). Three-dimensional isotropic magnetic resonance imaging can provide a reliable estimate of the native anterior cruciate ligament insertion site anatomy. Knee Surg Sports Traumatol Arthrosc.

[18] Defrate LE, Papannagari R, Gill TJ (2006). The 6 degrees of freedom kinematics of the knee after anterior cruciate ligament deficiency: an in vivo imaging analysis. Am J Sports Med.

[19] Tsai TY, Liow M, Peng Y (2018). In-vivo elongation of anterior and posterior cruciate ligament in bi-cruciate retaining total knee arthroplasty. J Orthop Res.

[20] Li G, Gil J, Kanamori A, Woo SL (1999). A validated three-dimensional computational model of a human knee joint. J Biomech Eng.

[21] Kozanek M, Hosseini A, Liu F (2009). Tibiofemoral kinematics and condylar motion during the stance phase of gait. J Biomech.

[22] Dimitriou D, Zou D, Wang Z, Tsai TY, Helmy N (2021). Anterior root of lateral meniscus and medial tibial spine are reliable intraoperative landmarks for the tibial footprint of anterior cruciate ligament. Knee Surg Sports Traumatol Arthrosc.

[23] Bland JM, Altman DG (1995). Comparing methods of measurement: why plotting difference against standard method is misleading. Lancet.

[24] Musahl V, Engler ID, Nazzal EM (2022). Current trends in the anterior cruciate ligament part II: evaluation, surgical technique, prevention, and rehabilitation. Knee Surg Sports Traumatol Arthrosc.

[25] Bedi A, Altchek DW (2009). The “Footprint” anterior cruciate ligament technique: an anatomic approach to anterior cruciate ligament reconstruction. Arthroscopy.

[26] Hosseini A, Lodhia P, Van de Velde SK (2012). Tunnel position and graft orientation in failed anterior cruciate ligament reconstruction: a clinical and imaging analysis. Int Orthop.

[27] Edwards A, Bull AMJ, Amis AA (2007). The attachments of the anteromedial and posterolateral fibre bundles of the anterior cruciate ligament: Part 1: tibial attachment. Knee Surg Sports Traumatol Arthrosc.

[28] Zantop T, Petersen W, Sekiya JK, Musahl V, Fu FH (2006). Anterior cruciate ligament anatomy and function relating to anatomical reconstruction. Knee Surg Sports Traumatol Arthrosc.

[29] Duthon VB, Barea C, Abrassart S (2006). Anatomy of the anterior cruciate ligament. Knee Surg Sports Traumatol Arthrosc.

[30] Mall NA, Chalmers PN, Moric M (2014). Incidence and trends of anterior cruciate ligament reconstruction in the United States. Am J Sports Med.

[31] Schilaty ND, Bates NA, Sanders TL (2017). Incidence of second anterior cruciate ligament tears (1990–2000) and associated factors in a specific geographic locale. Am J Sports Med.

[32] Schilaty ND, Nagelli C, Bates NA (2017). Incidence of second anterior cruciate ligament tears and identification of associated risk factors from 2001 to 2010 using a geographic database. Orthop J Sports Med.

[33] Wang J, Ao YF (2001). Epidemiological study of anterior cruciate ligament injury. Chin J Sports Med.

[34] Ding ZM, Chen B (2020) Epidemiological study of anterior cruciate ligament injury in trauma population. J Binzhou Med Univ 43(1):27–29, 45

[35] Xu JH (2015). Clinical value of MRI in the diagnosis of anterior cruciate ligament injury. Chin Foreign Med Res.

[36] Kassam AM, Tillotson L, Schranz PJ, Mandalia VI (2015). The lateral meniscus as a guide to anatomical tibial tunnel placement during anterior cruciate ligament reconstruction. Open Orthop J.

[37] Zantop T, Wellmann M, Fu FH, Petersen W (2008). Tunnel positioning of anteromedial and posterolateral bundles in anatomic anterior cruciate ligament reconstruction: anatomic and radiographic findings. Am J Sports Med.

[38] Ferretti M, Doca D, Ingham SM, Cohen M, Fu FH (2012). Bony and soft tissue landmarks of the ACL tibial insertion site: an anatomical study. Knee Surg Sports Traumatol Arthrosc.

[39] Hutchinson MR, Bae TS (2001). Reproducibility of anatomic tibial landmarks for anterior cruciate ligament reconstructions. Am J Sports Med.

[40] LaPrade CM, Smith SD, Rasmussen MT (2015). Consequences of tibial tunnel reaming on the meniscal roots during cruciate ligament reconstruction in a cadaveric model, part 1: the anterior cruciate ligament. Am J Sports Med.

[41] Kodama Y, Furumatsu T, Miyazawa S (2017). Location of the tibial tunnel aperture affects extrusion of the lateral meniscus following reconstruction of the anterior cruciate ligament. J Orthop Res.

[42] Oka S, Schuhmacher P, Brehmer A (2016). Histological analysis of the tibial anterior cruciate ligament insertion. Knee Surg Sports Traumatol Arthrosc.

[43] Steineman BD, Moulton SG, Haut DT (2017). Overlap between anterior cruciate ligament and anterolateral meniscal root insertions: a scanning electron microscopy study. Am J Sports Med.

[44] LaPrade CM, Jansson KS, Dornan G (2014). Altered tibiofemoral contact mechanics due to lateral meniscus posterior horn root avulsions and radial tears can be restored with in situ pull-out suture repairs. J Bone Joint Surg Am.

[45] Steineman BD, LaPrade RF, Santangelo KS (2017). Early osteoarthritis after untreated anterior meniscal root tears: an in vivo animal study. Orthop J Sports Med.

[46] Watson JN, Wilson KJ, LaPrade CM (2015). Iatrogenic injury of the anterior meniscal root attachments following anterior cruciate ligament reconstruction tunnel reaming. Knee Surg Sports Traumatol Arthrosc.

[47] Oishi K, Sasaki E, Naraoka T (2019). Anatomical relationship between insertion sites, tunnel placement, and lateral meniscus anterior horn injury during single and double bundle anterior cruciate ligament reconstructions: a comparative macroscopic and histopathological evaluation in cadavers. J Orthop Sci.

[48] Scheffler SU, Maschewski K, Becker R, Asbach P (2018). In-vivo three-dimensional MR imaging of the intact anterior cruciate ligament shows a variable insertion pattern of the femoral and tibial footprints. Knee Surg Sports Traumatol Arthrosc.

[49] Tashiro Y, Lucidi GA, Gale T (2018). Anterior cruciate ligament tibial insertion site is elliptical or triangular shaped in healthy young adults: high-resolution 3-T MRI analysis. Knee Surg Sports Traumatol Arthrosc.

[50] Irrgang JJ, Tashman S, Patterson CG et al (2021) Anatomic single vs double-bundle ACL reconstruction: a randomized clinical trial—Part 1: clinical outcomes. Knee Surg Sports Traumatol Arthrosc. 29(8):2665–2675 10.1007/s00167-021-06585-w PMC8298248 33970295

[51] Bosco F, Giustra F, Crivellaro M (2023). Is augmentation the best solution in partial anterior cruciate ligament tears? A literature systematic review and meta-analysis. J Orthop.

[52] Bosco F, Giustra F, Giai VR (2022). Could anterior closed-wedge high tibial osteotomy be a viable option in patients with high posterior tibial slope who undergo anterior cruciate ligament reconstruction? A systematic review and meta-analysis. Eur J Orthop Surg Traumatol..

[53] McGuire DA, Hendricks SD, Sanders HM (1997). The relationship between anterior cruciate ligament reconstruction tibial tunnel location and the anterior aspect of the posterior cruciate ligament insertion. Arthroscopy.

[54] Loucas M, Loucas R, D'Ambrosi R, Hantes ME (2021). Clinical and radiological outcomes of anteromedial portal versus transtibial technique in ACL reconstruction: a systematic review. Orthop J Sports Med.

